# Imaging electrochemically synthesized Cu_2_O cubes and their morphological evolution under conditions relevant to CO_2_ electroreduction

**DOI:** 10.1038/s41467-020-17220-6

**Published:** 2020-07-13

**Authors:** Rosa M. Arán-Ais, Rubén Rizo, Philipp Grosse, Gerardo Algara-Siller, Kassiogé Dembélé, Milivoj Plodinec, Thomas Lunkenbein, See Wee Chee, Beatriz Roldan Cuenya

**Affiliations:** 1 Department of Interface Science, Fritz-Haber-Institute of the Max-Planck Society, 14195 Berlin, Germany; 2 Department of Inorganic Chemistry, Fritz-Haber-Institute of the Max-Planck Society, 14195 Berlin, Germany

**Keywords:** Electrocatalysis, Renewable energy, Electrochemistry, Imaging techniques

## Abstract

Copper is a widely studied catalyst material for the electrochemical conversion of carbon dioxide to valuable hydrocarbons. In particular, copper-based nanostructures expressing predominantly {100} facets have shown high selectivity toward ethylene formation, a desired reaction product. However, the stability of such tailored nanostructures under reaction conditions remains poorly understood. Here, using liquid cell transmission electron microscopy, we show the formation of cubic copper oxide particles from copper sulfate solutions during direct electrochemical synthesis and their subsequent morphological evolution in a carbon dioxide-saturated 0.1 M potassium bicarbonate solution under a reductive potential. Shape-selected synthesis of copper oxide cubes was achieved through: (1) the addition of chloride ions and (2) alternating the potentials within a narrow window where the deposited non-cubic particles dissolve, but cubic ones do not. Our results indicate that copper oxide cubes change their morphology rapidly under carbon dioxide electroreduction-relevant conditions, leading to an extensive re-structuring of the working electrode surface.

## Abstract (web summary)

Catalytic selectivity during carbon dioxide electroreduction can be tuned by using geometric copper-based catalysts. Here, the authors use liquid cell transmission electron microscopy to study the in situ synthesis and morphological evolution Cu_2_O cubes under carbon dioxide electroreduction conditions.

## Introduction

Metal nanoparticles (NPs) are important catalyst materials for various energy harvesting applications because of their unique structural and electronic properties^1–4^. Therefore, there has been significant research into ways to engineer the detailed structure of NPs in order to create electrocatalysts with improved activity and selectivity for various electrochemical reactions. In particular, designing an effective electrocatalyst for the selective reduction of CO_2_ (CO_2_RR) into valuable products, such as ethylene or ethanol^4–6^, is still an on-going challenge. CO_2_RR powered by renewable energy sources is an attractive strategy for the sustainable production of chemicals and fuels. In this reaction, it is known that Cu-based nanostructures with cubic morphology have increased selectivity toward the desired C_2+_ reaction products^4,7–9^. It has also been shown that the detailed morphological parameters of cubic NPs, such as size and surface structure, can be used to further control the selectivity and stability^7–9^. Nevertheless, we still lack the precise control needed to fine-tune the structure of these NPs and optimize their electrocatalytic properties, despite the range of synthesis methods we have at our disposal to create NPs of different size, shape and composition, because it is not always clear which synthesis parameter is responsible for a certain morphology^10^. Another key question related to the use of shape-selected NPs in catalytic applications is their morphological stability under reaction conditions and whether the favorable structural motifs that are expected to lead to specific activity and selectivity trends persist during electrocatalysis. This question can be addressed through in situ and *operando* microscopic observations.

The synthesis of cubic-shaped Cu NPs can be achieved by wet chemical methods^7,11,12^ or by electrodeposition^8,13,14^. In chemical synthesis, shape selection is often achieved via the addition of facet-stabilizing ligands^7,11^. It is also commonly accepted that chloride ions (Cl^−^) in the precursor solution encourage nanocube formation because they stabilize the {100} facets of Cu^15,16^. However, how such effects lead to cube formation during electrochemical synthesis is yet to be clarified. The electrochemical synthesis of shaped-controlled NPs through halide additives is interesting for a few reasons. First, this method generates well-dispersed, shape-controlled NPs directly anchored to the support without the need of organic surfactants to keep the shape. In contrast, colloidal chemical synthesis usually needs organic surfactants^7,17^ which might be strongly attached to the catalyst surface, leading to catalytic properties that might be altered with respect to those from the underlying metal. Second, these halide anion species can also play a role in modifying the CO_2_RR selectivity^18^. A better understanding of the shape-selection mechanism(s) during electrochemical synthesis will allow us to develop strategies to control the formation of Cu-based nanocubes for subsequent CO_2_RR applications.

Liquid cell transmission electron microscopy (LC-TEM) is a relatively new technique that allows us to directly image nanostructures in a liquid environment with the high spatial resolution of a TEM^19–21^. By integrating thin film electrodes into the cells, we can study the dynamic behavior of materials in an electrolyte and under applied potential^22–31^. Although LC-TEM has been previously used to investigate shape-control mechanisms during colloidal synthesis^32–34^, it also constitutes a promising approach for the in situ study of electrocatalyst stability under reaction conditions^30,31^. In electrochemical LC-TEM studies, the electrocatalysts are usually synthesized using wet chemistry and then drop-casted on the working electrode of the liquid cells (LCs). While nonuniform Cu electrodeposition has already been shown in liquid TEM cells^22,23,35–37^, the electrochemical synthesis of shape-controlled particles within LCs is more challenging, due to their small volume and nonstandard thin film electrodes, and has not yet been demonstrated.

In this study, we show that we can electrochemically deposit Cu_2_O cubes directly from an aqueous CuSO_4_ solution within the LCs, and follow their subsequent stability in a CO_2_-saturated KHCO_3_ electrolyte under electrochemical reduction conditions. We further demonstrate that the shape-selection is due to Cl^−^ ions encouraging the formation of Cu_2_O particles, together with the enhanced stability of the Cu_2_O {100} facets, which protect the cubic Cu_2_O particles from dissolution at mildly oxidative potentials. Subsequently, we illustrate that fast morphological changes take place in the electrodeposited particles under conditions relevant to CO_2_RR. The latter results have significant implications for our understanding of the catalytic structures that exist under the actual reaction conditions and determine the selectivity trends.

## Results

### Observation of cubic-shaped Cu_2_O particle growth

**Fig. 1:**
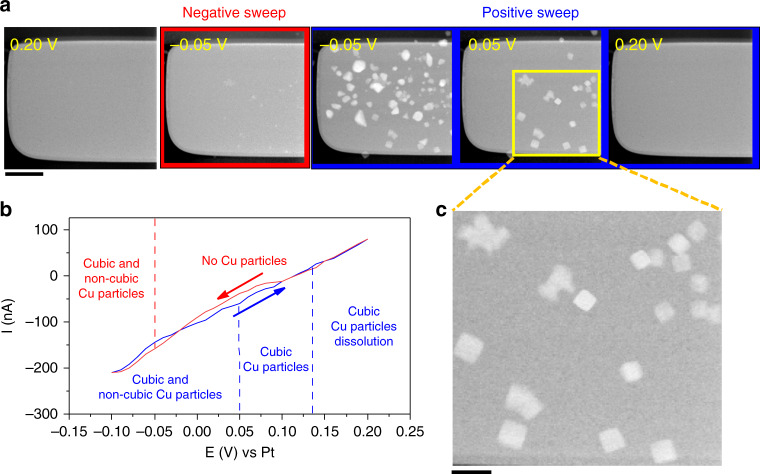
Electrochemical deposition of Cu_2_O cubes. **a** STEM images of the Pt working electrode at different potentials. The scale bar corresponds to 2 µm. **b** Cyclic voltammetry recorded at a scan rate of 5 mVs^−1^ in 5 mM CuSO_4_ + 5 mM KCl solution. **c** Expanded image extracted from the STEM image of the working electrode at −0.05 V during the positive-going scan clearly identifying Cu_2_O cubes on the electrode. The scale bar corresponds to 500 nm.

First, we discuss the synthesis of cubic-shaped Cu_2_O particles. A scheme of the cell setup, with a cross-section of the TEM LC and the top view of the chip which contains the three Pt electrodes (working electrode, reference electrode and counter electrode) is shown in Supplementary Fig. 1. The LC was first filled by flowing the precursor solution (aqueous 5 mM CuSO_4_ or 5 mM CuSO_4_ + 5 mM KCl solution)^8^ through the internal tubing prior to loading the holder into the TEM. Subsequently, the Pt working electrode was held at the open circuit potential (OCP). Figure 1a shows the image sequence that was captured with scanning transmission electron microscopy (STEM) and the cyclic voltammetry recorded a potential range of −0.10 and 0.20 V vs. a Pt pseudo-reference at 5 mVs^−1^, respectively. The first image clearly reveals that there is no copper deposited on the working electrode at the start of the experiment.

The second and third images show that particles nucleated and grew as the potential was ramped toward negative values. At these potentials, the shape and size distribution of the electrochemically deposited particles were inhomogeneous. As the potential was ramped to positive values once again, the non-cubic structures dissolved and only cubic particles remained on the working electrode (fourth image from Fig. 1a, c). Eventually, the cubes also dissolved as the potential approached 0.20 V. A movie describing the entire sequence is provided as Supplementary Movie 1. Supplementary Movies 2 and 3 show extended videos of the electrode under OCP at two magnifications, one at our typical imaging magnification and the second at a higher magnification. SEM images taken ex situ after synthesis also show the formation of cubes on other areas of the working electrode that had not been illuminated by the electron beam (Supplementary Fig. 2). These results indicate that there was no beam-induced nucleation at our imaging conditions and that the particle formation/dissolution were controlled by the applied potential.

Supplementary Figure 3 shows electron diffraction patterns collected in situ from the particles deposited on the working electrode. These results suggest that the as-deposited particles are a mixture of metallic copper and copper oxide. We also performed ex situ characterization of similar cubes deposited on a TEM grid with lacey carbon support (Supplementary Fig. 4). High-resolution TEM images show that the cubes are predominantly Cu_2_O with faint fringes that may be attributed to Cu (200) or CuCl (1 0 −1 4). Energy dispersive X-ray (EDX) spectroscopy analysis of one of such cubes indicates an overall composition of 61% Cu, 31% O, and 5% Cl. The mapping results also suggest that there is segregation of Cl within the particle, where higher intensity of the Cl signal is seen in the middle of the cube and a square band within the cube. These areas of segregated Cl also correspond to darker contrast in the STEM image and lower intensity in the Cu map. These results are in agreement with the cube composition previously reported^8^.

**Fig. 2:**
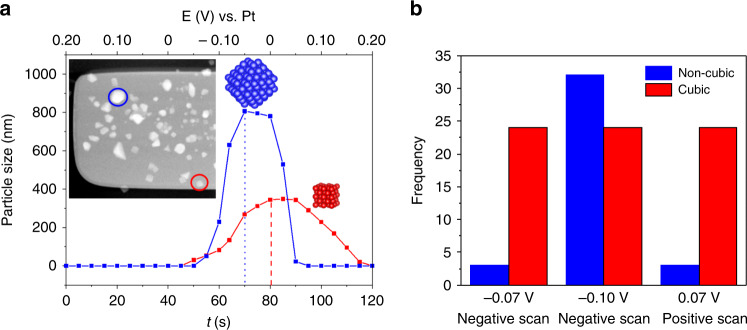
Potential-induced changes in particle size and morphology. **a** Non-cubic (blue solid line) and cubic (red solid line) particle size evolution of the selected particles marked in the STEM image with a circle of the corresponding color as a function of time and applied potential. **b** Evolution of the frequency of non-cubic and cubic particles deposited on the working electrode with the applied potential.

Figure 2a describes the size evolution of a Cu_2_O cube (red line) and a non-cubic (blue line) particle during the cyclic voltammetry in detail. Cu_2_O cubes started to form on the working electrode at −0.05 V during the negative-going scan. These cubes reach a maximum size of ~345 nm at 0.03 V and start dissolving at 0.07 V during the positive-going scan, and a potential of 0.20 V is required to completely dissolve them. In contrast, the growth of non-cubic particles started at −0.08 V during the negative-going scan and reached a maximum particle size of ~800 nm at −0.05 V during the positive-going scan. After ramping to more positive potentials, the non-cubic structures decrease in size rapidly and are completely etched from the working electrode surface at 0.05 V, a potential that is lower than the one needed to remove the cubic particles. Thus, Cu_2_O cubes were not only deposited at a lower overpotential than the non-cubic particles, but they were also stable over a broader potential range.

The populations of cubic and non-cubic particles at three different potentials are compared in Fig. 2b. These data confirm that the behavior described in Fig. 2a for a single particle is consistent across all the particles on the working electrode. Even though more non-cubic particles were formed during the negative-going scan, they also dissolved earlier when the potential reached positive values. Supplementary Figure 5 shows another image sequence from a different experiment where the same behavior was observed. It should be mentioned here that the potentials at which particles could be deposited and dissolved can vary between LCs, presumably due to differences in the properties of the thin-film pseudo-Pt reference electrodes and the ohmic drop across the length of the working electrode. Nevertheless, the Cu_2_O cubes are consistently more stable than the non-cubic particles across all experiments. The stabilization of Cu_2_O (100) facets by Cl^−^ during electrodeposition has been previously reported^16^. The enhanced stability of our Cl-rich Cu_2_O(100) cubes against oxidative dissolution is assigned to the shorter length of the Cu–O bond on the {100} plane as compared to {111} and {110}, leading to a stronger bond^38^. Our observations are in agreement with these previous findings^38,39^.

**Fig. 3:**
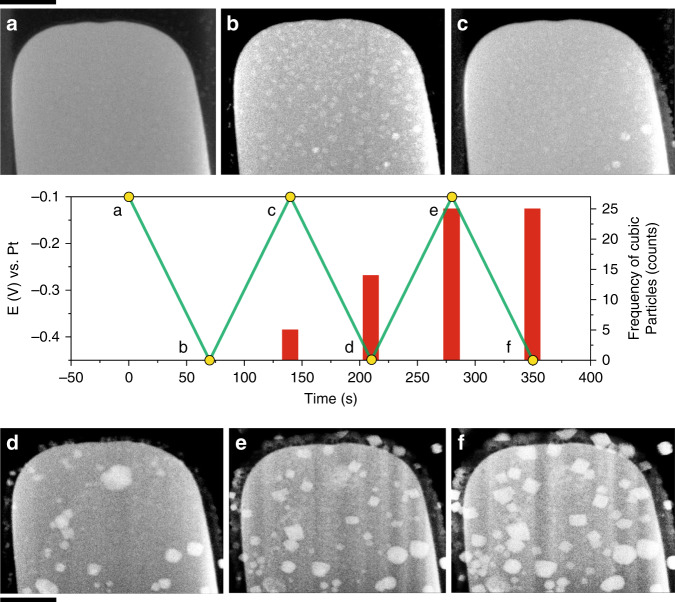
Number of cubic particles observed with potential cycling. Potential (green line) and frequency of cubic particles (red columns) as a function of experimental time during electrochemical cycling. The images labeled as **a**–**f** correspond to STEM images of the working electrode acquired at different potentials as indicated by the yellow circles in the plot. The vertical bars indicate the number of cubic-shaped particles observed at each point. The scale bars correspond to 1 µm.

The number of Cu_2_O cubes also increased upon repeatedly cycling the potential. Figure 3 shows the results from one such experiment where we cycled the potentials at an upper potential where the particles did not fully dissolve from the working electrode (Supplementary Movie 4). Again, particles with different shapes nucleated and grew during the negative-going scan of the first cycle. The cubes largely survived the positive-going scans. In this case, the number and size of cubic particles increased with successive cycles (images in Fig. 3), as new cubic architectures were nucleated and the retained cubes grew larger. This behavior resulted in increasing shape-selection as the synthesis process continued.

### Influence of Cl^−^ ions on the particle morphology

In colloidal particle synthesis, it is considered that the addition of Cl^−^ ions leads to shape-selection toward cubic NPs via kinetic control and oxidative dissolution of non-cubic, twinned NPs^40–42^. Kim et al.^43^ discussed that Cl^−^ favors the formation of cubic Cu_2_O particles during colloidal synthesis by slowing down the nucleation and growth rate. Chloride ions in the precursor solution also encourage nanocube formation by stabilizing the {100} facets of Cu^15,16^ and Cu_2_O^38^. The shape of Cu nanocrystals in a solution containing different ions (Na^+^, NH_4_^+^, SO_4_^2^^−^, Cl^−^, dodecyl sulfate) was also investigated^15^. Interestingly, regardless of the initially exposed crystal facets, only {100} planes remained after exposing these crystals to Cl^−^-containing solutions for a few minutes, leading to cubic shapes. Moreover, the electrochemical nanostructuring of Cu foils into cubic facets was observed in the presence of Cl^−^, while flatter larger morphologies and needle-like structures were obtained when cycling in the presence of Br^−^ and I^−^, respectively^44^. We also showed recently^8^ that Cu cubes can be grown by electrodeposition on a carbon substrate (HOPG) using CuSO_4_ as Cu source to create NPs and KCl to reshape them. However, the former study only contained ex situ atomic force microscopy (AFM) data of the final state of the samples after the synthesis, while here we present an in situ microscopy study that allows us to follow in detail the electrochemical parameters influencing the formation of the cubes, and even more importantly, the potential regimes in which the cubic shapes are most stable. In addition, by employing a different support material (Pt), we are able to demonstrate now that the previously described synthesis is not only restricted to carbon-based substrates.

**Fig. 4:**
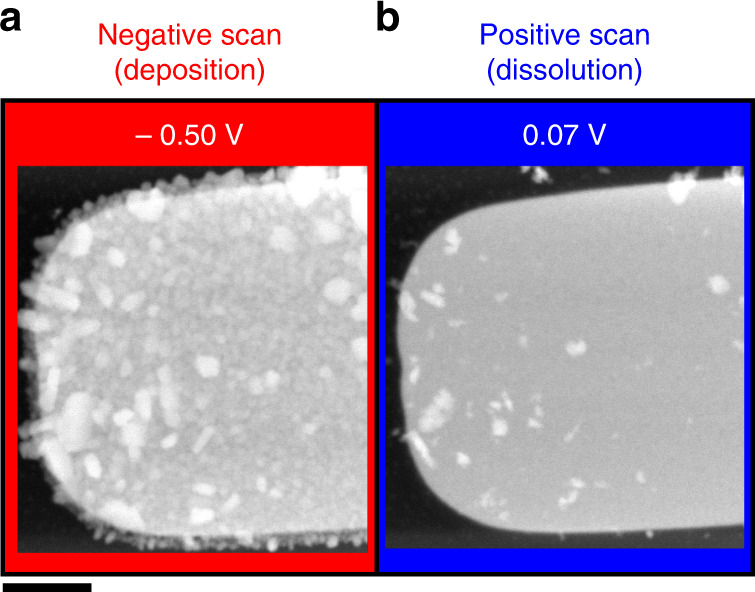
Particle growth in the absence of Cl^−^ ions. STEM images of the working electrode at different potentials during cyclic voltammetry recorded at a scan rate of 5 mVs^−1^ in a 5 mM CuSO_4_ solution. The scale bar corresponds to 2 µm.

The question now is if the presence of Cl^−^ ions in fact influences the particle morphology during nucleation. The image sequence in Fig. 4 of particles grown using a 5 mM CuSO_4_ solution with no KCl added (Supplementary Movie 5), demonstrates that no cubic particles are formed in the absence of Cl^−^ ions. Hence, it suggests that KCl is in fact necessary for the shape-selection toward Cu_2_O cubic particles. As further control experiments to verify the role of Cl^−^ ions, we performed benchtop electrodeposition on glassy carbon supports with 5 mM CuSO_4_ solutions containing either 5 mM KCl, 5 mM NaCl, or 5 mM HCl (Supplementary Fig. 6) as additive to check if the cations had any effect on the particle morphology. Cubes were seen in both KCl and NaCl, but not when HCl was used. The absence of cubes in the HCl experiment can be rationalized from the Pourbaix diagram of Cu in aqueous solutions containing Cl^−45^, where the Cu_2_O phase is destabilized at the lower pH, thus leading to the loss of the cubic morphology. Hence, these results confirm that the specific alkali metal cations had no influence on the electrodeposition and that the cubes are nucleated from Cu_2_O.

More importantly, our in situ observations show that the presence of Cl^−^ ions does not directly lead to the solely nucleation of cubic particles. Instead, mixed morphologies are formed during the initial nucleation stage, and a second dissolution mechanism and the optimum applied potential are required to refine the particle shape distribution. This adds valuable insight for the further optimization of the synthesis recipes towards catalysts with narrow size and shape distributions.

### Dynamical changes under reducing conditions in 0.1 M KHCO_3_

**Fig. 5:**
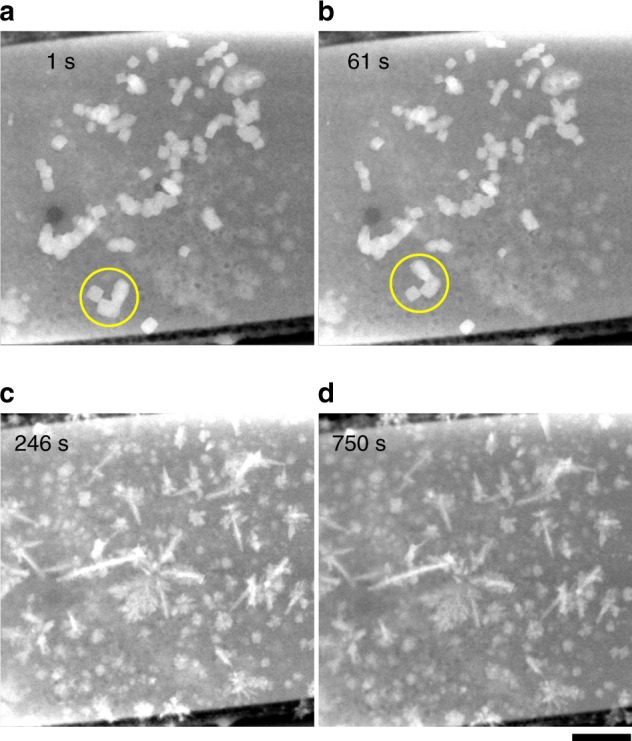
Evolution of Cu_2_O cubes during reducing conditions. STEM images of the working electrode at different experimental times, **a** *t* = 1 s, **b** *t* = 61 s, **c** *t* = 246 s, and **d** *t* = 750 s, acquired while keeping the potential at −0.7 V in a CO_2_-saturated 0.1 M KHCO_3_ solution. It can also be seen that many cubes have started to reduce in size. A cluster of cubic particles that moved between *t* = 1 s and *t* = 61 s is highlighted with a yellow circle. After *t* = 245 s, no further changes in the nanostructure morphology were apparent. The scale bar corresponds to 1 µm.

We further exposed these as-synthesized cubes directly to reducing conditions where CO_2_ electroreduction takes place and were able to monitor drastic changes of the catalyst structure in real time. Figure 5 shows one set of results from these experiments. Here, we exchanged the electrolyte by flowing CO_2_-saturated 0.1 M KHCO_3_ through the cell for at least 30 min while keeping the potential of the working electrode at −0.1 V to avoid particle dissolution at OCP. It can be seen from Supplementary Fig. 7 that the cubes did not exhibit significant changes during the fluid exchange. Then, a potential of −0.7 V was applied to the working electrode, which led to dynamical morphological changes taking place on the working electrode within the cell (Fig. 5, Supplementary Movie 6). Between *t* = 1 s and 61 s, we can see that some of the cubes were not strongly attached to the surface and they started moving across the electrode surface when the potential was applied. At the same time, we see concurrent size reduction of the original cubes and the formation of new copper structures on the working electrode, some in the form of dendritic structures (*t* = 61 s to 246 s). It should be noted here that the potentials reported may be up to ~0.2 V off from the applied values due to the electrochemical cell configuration of these in situ TEM studies and the pseudo-Pt reference electrode.

The morphological changes directly observed here in the Cu_2_O particles by in situ LC-TEM under CO_2_RR conditions are expected to lead to changes in the product selectivity, as previously discussed based on ex situ SEM data^8,9,46^. The progressive loss of the (100) facets leads to an increase in H_2_ production at the expense of the CO_2_RR products. However, a higher stability and C2–C3 product yield was observed during CO_2_RR for Cu NCs electrochemically grown on Cu foils^9^. Even more importantly, the highest ethylene yield was obtained when the Cu NCs/Cu samples were pretreated with an oxygen plasma which lead to a drastic change in the structure and the loss of the cubic shape after reaction, while the same samples pretreated in Ar-plasma retained the cubic structure but resulted in a significantly lower C2–C3 product selectivity. In general, the loss of Cu(100) facets is correlated with a decrease in the yield of ethylene^9,44,47^, although other factors such as the content of subsurface oxygen or Cu(I) species have also been mentioned to play a role^9,48^. Unfortunately, correlating the observed structural transformations with their impact on reaction product selectivity is still a nontrivial technical challenge for LC-TEM, and outside the scope of this work.

Our in situ TEM observations of highly dynamic Cu catalysts are consistent with a recent study using identical location SEM also reporting significant restructuring of Cu NP catalysts within minutes of applying the potential to initiate CO_2_RR^49^. The formation of dendritic structures has been attributed to the simultaneous dissolution and redeposition of Cu under CO_2_ reduction conditions^50^. A similar mechanism may be at play in our experiments. We speculate that the Cu species that end up forming dendrites originate from material (Cu atoms and small Cu clusters not visible during the in situ TEM) that was deposited on the working electrode during in situ electrodeposition but not nucleated as cubes. These Cu species on the electrode surface become mobile when the potential is applied, leading to a re-distribution of the as-deposited Cu and the consequent dendritic nanostructure growth. In other areas of the working electrode, we can also observe a size reduction in the original as-synthesized cubes, similar to that previously reported via electrochemical atomic force microscopy for Cu_2_O cubes grown on graphite, albeit at much higher rates. The fact that the size reduction of the cubes and additional particle growth occurred at the same time in our experiments also suggests that the behavior we observe is not due to the reduction of residual Cu ions in solution. The differences between the dynamic morphological changes observed for the electrochemically grown Cu cubes versus the former study^8^ might be due to the different supports used, graphite (HOPG) vs. Pt, as well as the different configurations of the electrochemical cells employed. The cell geometry can influence the flow rate of the electrolyte and determine the local chemical concentration and potential gradients at the working electrode. Our future work will focus on fine tuning our synthesis protocol such that size-selected cubic-like structures are also generated in situ for more detailed studies looking at the effects of both shape and size in these catalysts during CO_2_RR.

We also briefly address the possibility of electron beam-induced artifacts in the particle growth and CO_2_ electroreduction experiments in the bicarbonate electrolyte. We have used a low electron dose rate of ~ 20 e^−^ nm^−1^s^−1^ in these experiments and thus, such effects are not apparent. Supplementary Fig. 8 shows that the morphological changes seen in the Cu_2_O structures in Fig. 5 and in Supplementary Movie 6 were not due to artifacts from sustained imaging with the electron beam. In this control experiment, we imaged the as-deposited particles for 750 s after electrolyte exchange to CO_2_-saturated 0.1 M KHCO_3_ before applying the −0.7 V potential. As expected, and regardless of the prior electron beam irradiation, Cu restructuring and dendrite growth was only seen when the potential was applied.

## Discussion

The present study emphasizes the advantages of using in situ LC-TEM for monitoring the nanoscale evolution of electrocatalysts at timescales of seconds. Nevertheless, the resolution degradation associated with imaging through a liquid^19^ makes it difficult to capture small structural changes in the in situ image sequences. Furthermore, key technical challenges still remain to be solved before one can directly correlate the morphological modifications observed with the catalytic performance. These include the minute liquid volumes held in the TEM cells, the small size of the electrodes in the microfabricated set-ups, the low catalysts loading, and the need for product detection instrumentation with faster time–response and improved sensitivity. Nevertheless, we expect that future improvements of the experimental set-ups will eventually provide us with the ability to relate the evolution of the structure of an electrocatalyst with changes in its activity, selectivity and stability, providing in depth insight for rational electrocatalyst design.

In conclusion, we demonstrated that we can directly synthesize shape-controlled catalyst particles on the working electrode of a microfluidic TEM LC and follow their subsequent dynamics under reaction conditions. In particular, the in situ TEM observations during synthesis show that the addition of Cl^−^ ions into the precursor CuSO_4_ aqueous solution induces the nucleation of cubic structures among non-cubic particles and stabilizes these cubes against dissolution. The cubes require a lower overpotential to be deposited and are stable over a larger range of potentials. Additional potential cycles lead to an increase in the number of cubic particles and growth of the cubes deposited in the previous cycles. These experiments provide new insights into the electrodeposition parameters required to custom-tune the synthesis of size- and shape-selected NPs. Moreover, the ability to directly deposit cubic particles on the working electrode of a LC provides a straightforward way to create these electrocatalysts for subsequent in situ studies of their behavior during CO_2_RR.

The observation of the in situ synthesized Cu_2_O cubes under conditions relevant to the electrochemical reduction of CO_2_ also revealed that fast and extensive restructuring takes place within a few minutes of applying a reductive potential in a CO_2_-saturated bicarbonate solution. The results shown here emphasize the highly dynamic nature of electrocatalysts under reaction conditions, including their mobility and their ability to serve as nucleation sites for the growth of Cu dendrites. Finally, this study highlights the advantages of using in situ LC-TEM for the mechanistic investigation of the parameters affecting the electrochemical synthesis of catalytically relevant materials and the excellent possibilities of monitoring their performance in situ during an electrocatalytic process.

## Methods

### Chemicals and materials

Potassium chloride (KCl, 99.0–100.5%), copper sulfate pentahydrate (CuSO_4_·5H_2_O, 99.995%) and potassium bicarbonate (KHCO_3_, 99.9%) were obtained from Sigma-Aldrich. All chemicals were used as received.

### LC-TEM experiments

For these experiments, a liquid biasing DENSsolution Stream holder and a flow cell chip equipped with a silicon nitride window and three Pt electrodes were employed. An overview of the electrodes is displayed in Supplementary Fig. 1. A pressure based-liquid pump was used to flow the working electrolyte (CuSO_4_ + KCl and/or CuSO_4_ solutions) inside the microfluidic cell and a CompactStat potentiostat (Ivium Technologies, Netherlands) was connected to the holder for conducting the electrochemical deposition of copper particles on the working electrode. The LC-TEM holder was leak tested in a vacuum pump station provided by DENSsolutions.

The in situ microscopy was performed using a FEI Titan 80–300 transmission electron microscope operated in scanning (STEM) mode at 300 kV. Electron beam conditions were selected to optimize the imaging conditions while maintaining a low overall electron dose rate of no more than 20 e^−^ nm^−2^ s^−1^ during image acquisition. At these dose rates, we did not see obvious effects of the electron beam on the experiment, dynamic changes were only observed during the application of the potential. The movie acquisition rate was 1 frame per second.

Cu_2_O cubic particles were grown in a 5 mM CuSO_4_ + 5 mM KCl solution by electrochemical cycling in a potential window of 0.3 V between the starting points of the Cu reduction and oxidation processes at a sweep rate of 5 mVs^−1^. The limits of the scan were determined in situ by finding the potentials at which extensive Cu deposition/dissolution were observed on the working electrode. Such approach was implemented because there were shifts in the potentials between in situ experiments of up to ~200 mV, which we partially assigned to the microfluidic electrochemical cell configuration and the Pt pseudo-reference electrode. Hence, the potentials we used for the in situ imaging show small deviations from the potentials we employed in the benchtop set-ups. Similar deviations have also been reported by other researchers^51^.

### Ex situ SEM and TEM imaging

A Thermo Fisher Apreo scanning electron microscope operated was used to collect ex situ images of the chip. TEM, HRTEM, and EDX elemental mapping of the cubic particles deposited on the TEM grid were acquired on a high resolution analytical transmission electron microscope (JEOL, JEM-2800).

## Supplementary information

Supplementary Information

Supplementary Movie 1

Supplementary Movie 2

Supplementary Movie 3

Supplementary Movie 4

Supplementary Movie 5

Supplementary Movie 6

## Data Availability

The data that support the plots within this paper and other findings of this study are available from the corresponding authors upon reasonable request.
