# Supplementary material for: Imaging electrochemically synthesized Cu2O cubes and their morphological evolution under conditions relevant to CO2 electroreduction
Source: Nat Commun. 2020 Jul 13;11:3489. doi: 10.1038/s41467-020-17220-6 (PMC7359295; doi:10.1038/s41467-020-17220-6)
Supplement: Supplementary file 1 — Supplementary Information [file 41467_2020_17220_MOESM1_ESM.pdf]

Supporting information

Imaging electrochemically synthesized Cu_2O cubes and their morphological evolution under conditions relevant to CO_2 electroreduction

Arán-Ais *et al.*

Supplementary Figures

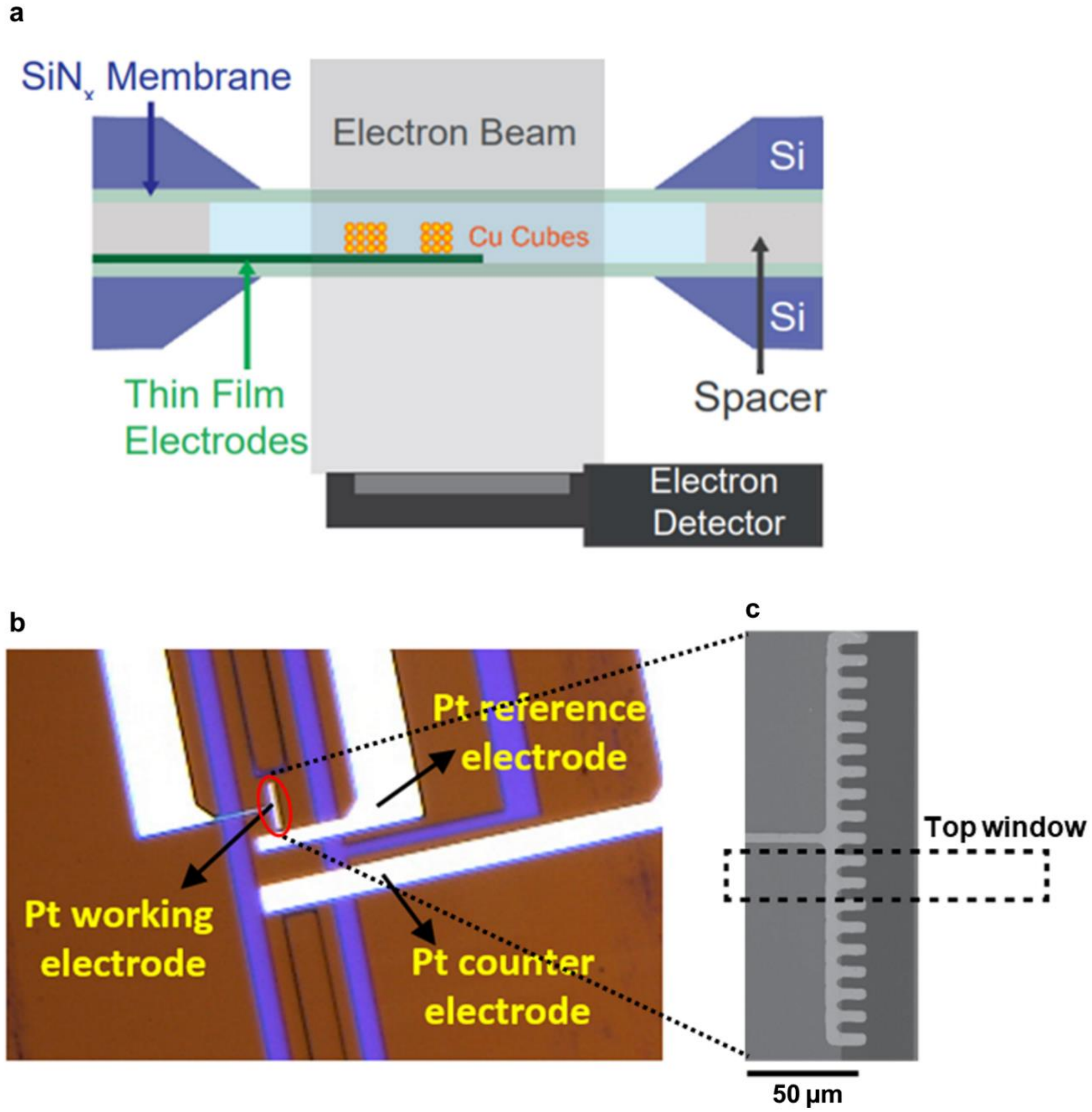

Supplementary Figure 1. Overview of an electrochemical cell setup, with **a,** a cross section of the TEM liquid cell on the top and **b,** view of the electrodes on the electrochemistry chip from DENS solutions on the bottom. As shown in the schematic, the liquid cell isolates the electrolyte from the vacuum of the TEM column by enclosing the liquid between two electron-transparent silicon nitride membrane windows. **c,** SEM image of the working electrode in the liquid cell. The dashed box denotes the approximate size of the top window.

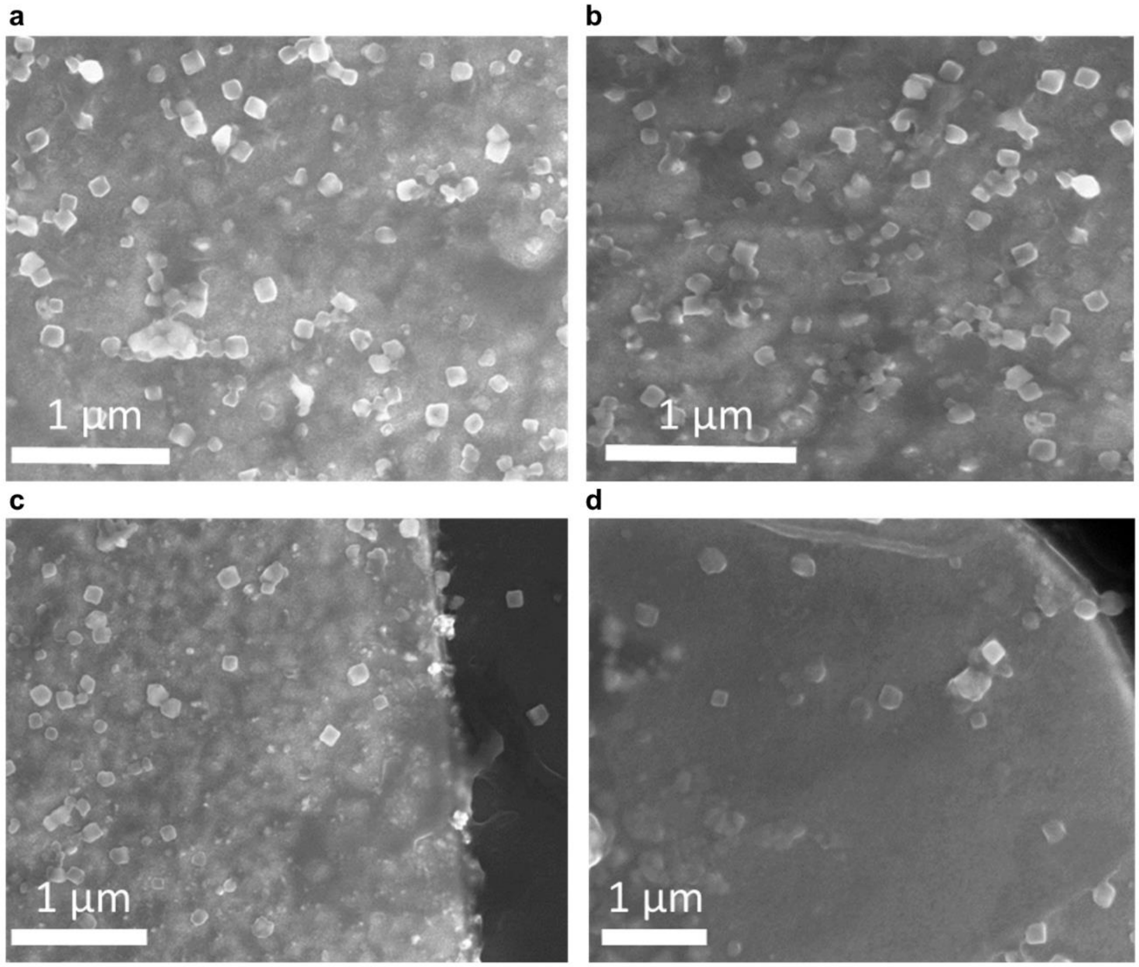

Supplementary Figure 2. a-d, *Ex situ* SEM images of the working electrodes in an experiment where we carried out the synthesis outside the TEM, i.e., the samples were not exposed to the electron beam.

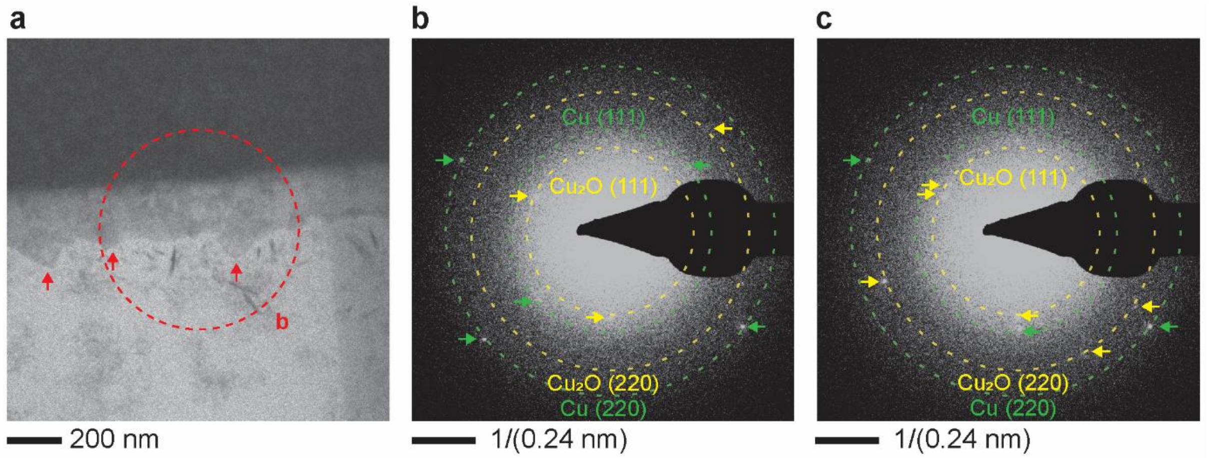

Supplementary Figure 3. *In situ* electron diffraction patterns of electrodeposited Cu particles. a, Cubic-shaped Cu particles deposited on the edge of the working electrode. The selected area used for the electron diffraction is outlined with a red dashed circle. Red arrows denote the cubic particles. **b,** Electron diffraction pattern from the area selected in (a). **c,** Electron diffraction pattern from a different area further along the working electrode out of the field of view of (a).

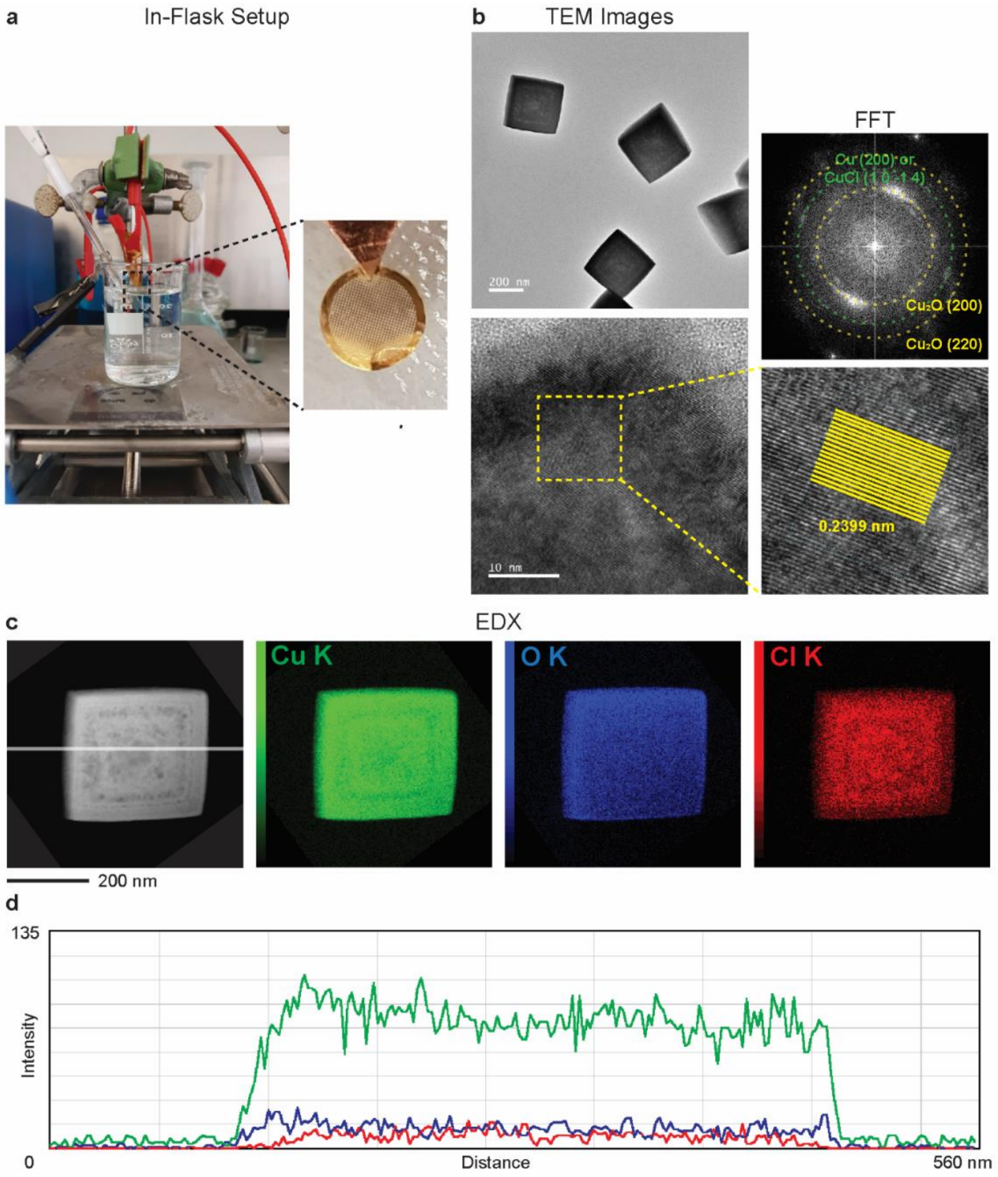

Supplementary Figure 4. *Ex situ* characterization of electrodeposited Cu_2O particles. a, Experimental setup for the electrodeposition of Cu_2O cubes on different substrates, consisting on a beaker containing the precursor solution, a Pt counter electrode, a Ag/AgCl reference electrode and a TEM grid acting as working electrode. **b,** TEM, HRTEM and Fast Fourier Transform (FFT) of the cubic particles deposited on the TEM grid. **c,** STEM image and EDX elemental mapping for copper (green), oxygen (blue) and chlorine (red). The overall composition of the particle from EDX is 61% Cu, 31% O and 5% Cl. The Cl K map suggests there is segregation of Cl within the particle, with more Cl in the middle of the cube and a square band within the cube. These areas of segregated Cl correspond to darker contrast in the STEM image and lower intensity in the Cu map. The line in the STEM image denotes the location of the line profile displayed in (d). **d,** Line profile of characteristic X-ray intensity across the particle. The colors are the same as in (c).

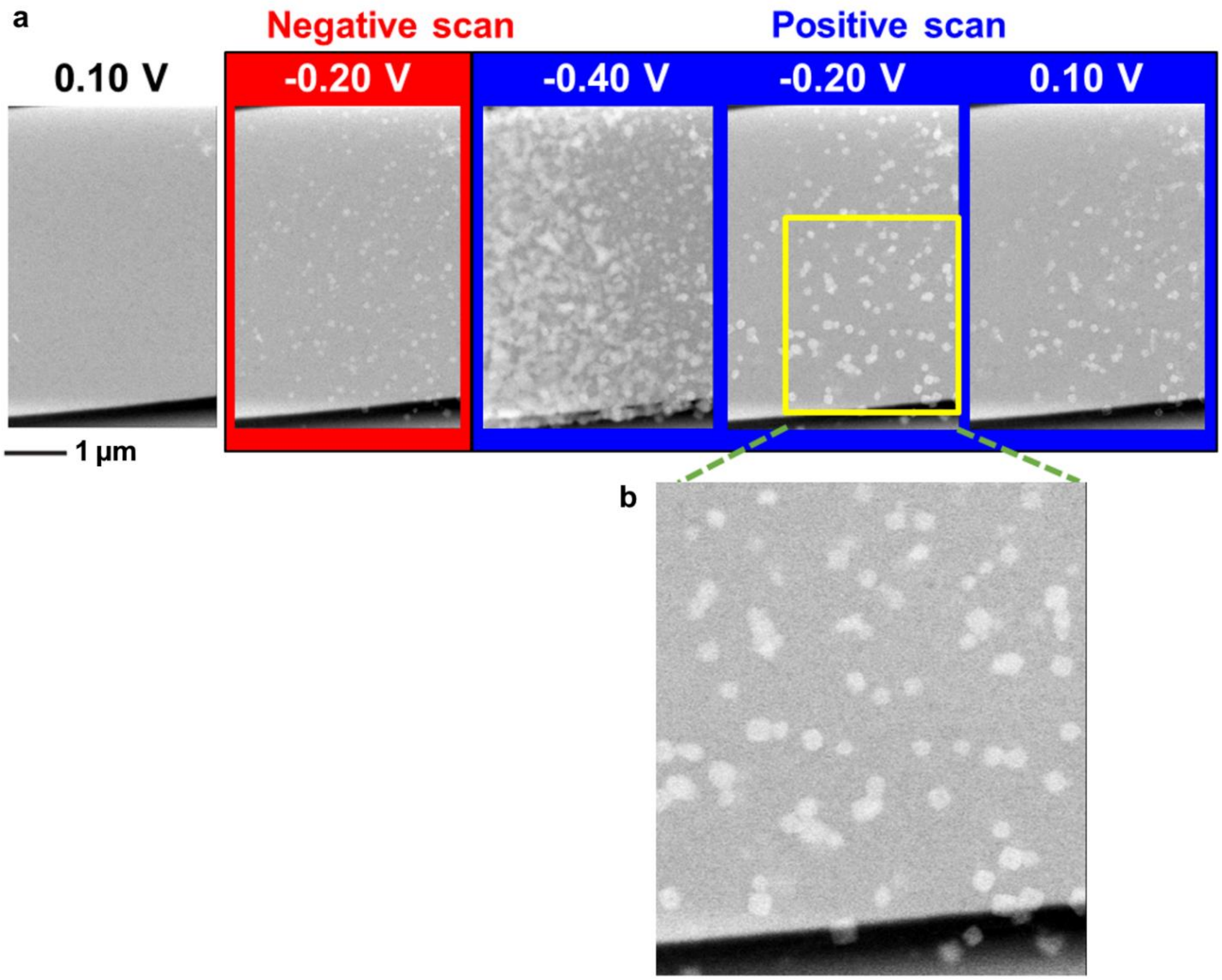

Supplementary Figure 5. a, STEM images of the working electrode at different potentials during cyclic voltammetry recorded at a scan rate of 5 mVs^{-1} in 5 mM $CuSO_4$ + 5 mM KCl solution. These images correspond to a different experiment (different pair of chips assembly) with respect to the one presented in the main paper but with similar results. **b,** Magnified area of the -0.20 V image showing the cubic-shaped particles.

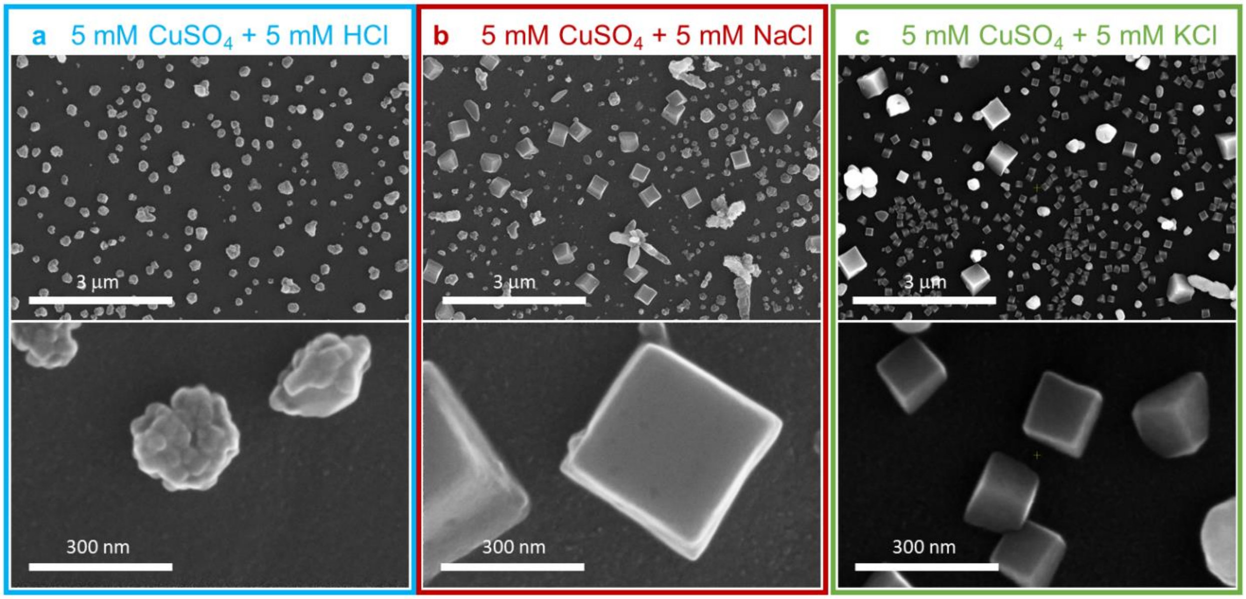

Supplementary Figure 6. *Ex situ* SEM images of Cu structures electrodeposited on a glassy carbon electrode using **a,** 5 mM $CuSO_4$+ 5 mM HCl; **b,** 5 mM $CuSO_4$+ 5 mM NaCl and **c,** 5 mM $CuSO_4$+ 5 mM KCl solutions. The particles were electrodeposited by sweeping the potential between -0.5 V and -0.2 V vs Ag/AgCl.

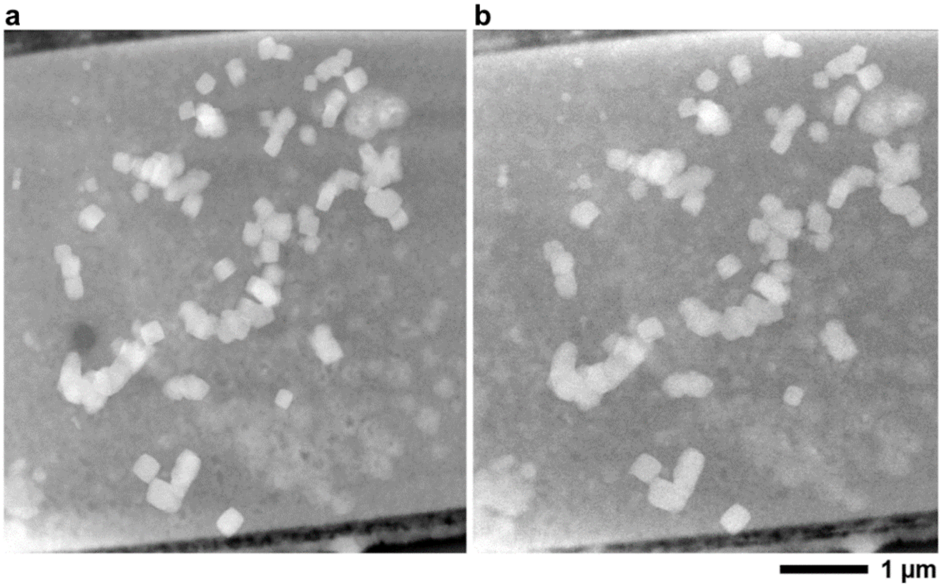

Supplementary Figure 7. *In situ* TEM images comparing the electrodeposited Cu cubes described in Figure 5. **a,** before and **b,** after the flow of a CO_2-saturated 0.1 M $KHCO_3$, but before the applications of -0.7 V.

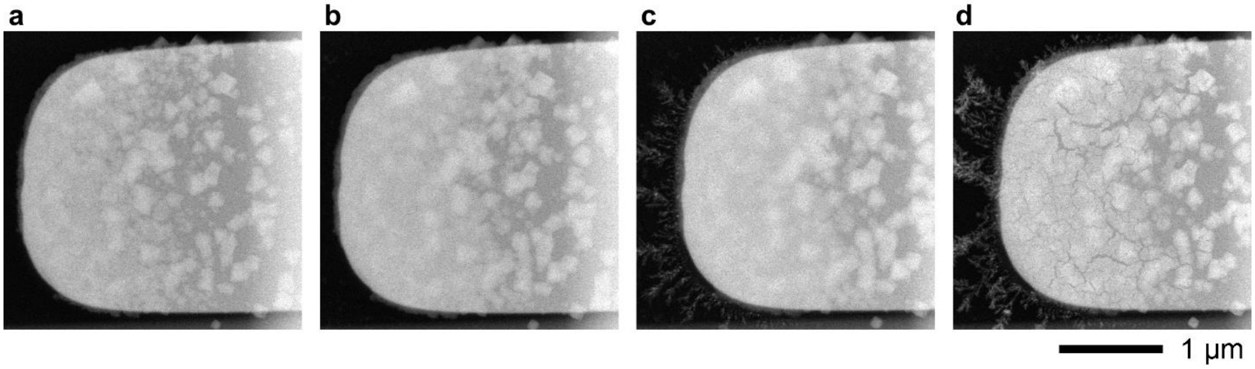

Supplementary Figure 8. An image sequence from a control experiment where we check that the re-structuring shown in Figure 5 of the main text was not caused by artifacts related to the electron beam. The electrodeposited cubic-like Cu_2O structures were imaged in a CO_2-saturated 0.1M $KHCO_3$ solution for 750 s before the application of the -0.7 V potential. Images of the working electrode after **a,** electrodeposition and electrolyte exchange, **b,** 750 s of imaging, **c,** 5 s after applying -0.7 V against the pseudo-Pt reference, and **d,** 58 s after applying -0.7 V.
